# Genetic risk score associations for myocardial infarction are comparable in persons with and without rheumatoid arthritis: the population-based HUNT study

**DOI:** 10.1038/s41598-020-77432-0

**Published:** 2020-11-24

**Authors:** S. Rostami, M. Hoff, H. Dalen, K. Hveem, V. Videm

**Affiliations:** 1 Department of Clinical and Molecular Medicine, St. Olavs Hospital, NTNU - Norwegian University of Science and Technology, Lab Center 3 East, 7006 Trondheim, Norway; 2 Department of Neuromedicine and Movement Science, NTNU - Norwegian University of Science and Technology, Trondheim, Norway; 3 Department of Public Health and Nursing, NTNU - Norwegian University of Science and Technology, Trondheim, Norway; 4 Department of Rheumatology, St. Olavs University Hospital, Trondheim, Norway; 5 Department of Circulation and Medical Imaging, NTNU - Norwegian University of Science and Technology, Trondheim, Norway; 6 Clinic of Cardiology, St. Olavs Hospital, Trondheim University Hospital, Trondheim, Norway; 7 Levanger Hospital, Nord-Trøndelag Hospital Trust, Levanger, Norway; 8 KG Jebsen Center for Genetic Epidemiology, NTNU - Norwegian University of Science and Technology, Trondheim, Norway; 9 Department of Immunology and Transfusion Medicine, St. Olavs University Hospital, Trondheim, Norway

**Keywords:** Epidemiology, Outcomes research, Genetics, Cardiology, Rheumatology

## Abstract

Persons with rheumatoid arthritis (RA) have increased risk of myocardial infarction (MI). Overlapping associations with MI of weighted genetic risk scores (wGRS) for coronary artery disease (CAD) and RA is unknown in a population-based setting. Data from the prospective Nord-Trøndelag Health Study (HUNT2: 1995–1997 and HUNT3: 2006–2008) were used. wGRS added each participant’s carriage of all risk variants weighted by the coefficient from published association studies. Published wGRS for CAD and RA were analysed in Cox regression with MI as outcome, age as analysis time, and censoring at the first MI, death, or 31.12.2017. 2609 of 61,465 participants developed MI during follow-up (mean 17.7 years). The best-fitting wGRS for CAD and RA included 157 and 27 single-nucleotide polymorphisms, respectively. In multivariable analysis including traditional CAD risk factors, the CAD wGRS was associated with MI [hazard ratio = 1.23 (95% CI 1.18–1.27) for each SD increase, *p* < 0.0001] in RA patients (n = 433) and controls. The RA wGRS was not significant (*p* = 0.06). Independently from traditional risk factors, a CAD wGRS was significantly associated with the risk for MI in RA patients and controls, whereas an RA wGRS was not. The captured genetic risk for RA contributed little to the risk of MI.

## Introduction

Rheumatoid arthritis (RA) is a chronic autoimmune inflammatory disorder that affects joints and is associated with several comorbidities and accelerated death^1^. Myocardial infarction (MI) is the main undesirable outcome of coronary artery disease (CAD)^2^. There is extensive evidence for the association of RA with CAD from epidemiological and pathophysiological studies, suggesting a ~ 70% increased risk of MI among RA patients compared to the general population^3, 4^.

Diabetes, hypertension, dyslipidemia, obesity, smoking, age, and male sex are among the traditional risk factors for CAD. Smoking, older age, and female sex are risk factors for RA. Thus, the traditional CAD-related risk factors may show different strengths of association when evaluated in RA patients versus the general population^5^. Nevertheless, these factors alone do not explain the increased risk of CAD among RA patients^6^, which may partly be attributed to RA characteristics such as increased disease activity and severity, systemic chronic inflammation, as well as mechanisms leading to or accelerating atherosclerosis^6, 7^.

A nation-wide prospective Swedish study among patients with incident RA and their siblings suggested that full siblings of seropositive RA patients were at an increased risk of acute coronary syndrome (ACS) compared to the general population even after adjustment for traditional CAD risk factors. This implies a shared susceptibility (genetic and/or environmental) between seropositive RA and ACS^8^. In genome-wide association studies, ~ 21% of CAD heritability is explained by 304 known genetic variants^9^.

CAD risk models which are mainly based on established traditional risk factors for the general population have comparatively lower performance in RA patients^10^. There have been attempts to improve CAD risk prediction by further incorporating RA status or RA characteristics into models for the general population, with contradictory results^10–12^. Several genetic risk scores have previously been developed for RA and CAD^13^. *HLA-DRB1* was suggested as useful for screening due to its association with increased risk of CAD mortality among RA patients^14^. However, none of the previous studies, to our knowledge, have evaluated whether the associations of MI with genetic risk scores (GRS) for CAD and RA have overlapping effects in a population-based setting including RA patients and controls.

The hypotheses of the present study were: (1) GRS for CAD from other general European populations are also associated with MI in the HUNT study, and (2) Due to overlapping genetic susceptibility for RA and CAD, the best-fitting GRS for RA from HUNT partly explains the increased risk of MI in RA patients. The main objective was to evaluate the overlap and associations with MI of published weighted GRS (wGRS) for CAD and a wGRS for RA from HUNT, adjusted for relevant traditional CAD risk factors, in the Norwegian HUNT cohort including RA patients and a large control group using a case/cohort design.

## Methods

**Figure 1:**
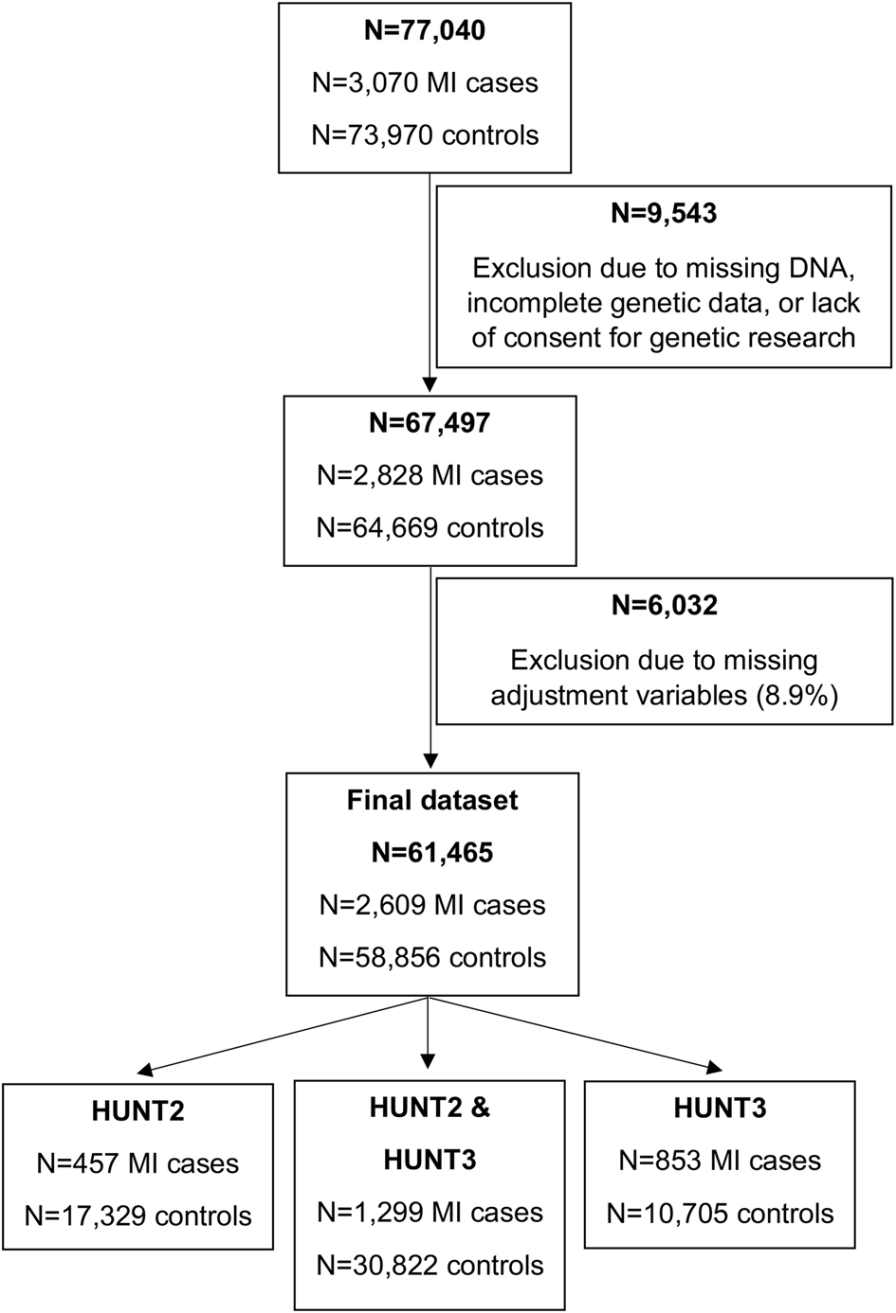
Inclusion and exclusion of study participants. Abbreviation: MI: myocardial infarction.

The population-based prospective cohort Nord-Trøndelag Health Study (HUNT) has been conducted among all adults ≥ 20 years in the Northern region of Trøndelag County, Norway since 1984^15^. The participation rate in HUNT2 was approximately 70% of those invited and the corresponding rate in HUNT3 was approximately 54%. Data collection was based on questionnaires, interviews, clinical examinations, as well as blood sampling in HUNT2-HUNT4. We used data from HUNT2 (1995–1997) and HUNT3 (2006–2008)^15^, and initially included a dataset of 578 patients with RA and a large cohort of controls (76,462) as previously described^13^ (Fig. 1). Genetic data were missing from 9542 participants (12.4%), the main reasons being missing DNA or no consent for genetic research. We considered that it would not be appropriate to perform multiple imputation of the GRS for these participants. Data for adjustment variables in the analyses as further described below were missing for 6032 participants (8.9%). With missingness < 10%, multiple imputation of these variables was not considered necessary. Thus, the final dataset consisted of participants with complete data for the relevant variables. Data from HUNT1 (1984–1986) could not be used because there were no questions regarding RA. The dataset was linked to the Myocardial Infarction Registry in the Nord-Trøndelag Health Trust that contains data collected retrospectively from the two regional hospitals (Levanger Hospital and Namsos Hospital) between 1995 and 2000 and prospectively since 2001. From 2012, data regarding MI were found by linkage to the National Norwegian Cardiovascular Disease Registry, which used the same data collection methods as during the previous years. Data were also linked to the National Cause of Death Registry to permit censoring due to death during the observation period. The Cardiovascular Disease Registry has a national completeness of approximately 92% and the Cause of Death Registry has a completeness of > 99%. Inclusion years used in the present study were from the start of HUNT2 (1995) to end of follow-up on 31.12.2017.

MI was diagnosed using contemporary guidelines from the European Society of Cardiology/American College of Cardiology^16^. We used RA diagnoses from the HUNT project HuLARS (**HU**NT **L**ongitudinal **A**nkylosing spondylitis and **R**heumatoid arthritis **S**tudy) as previously described^17^. The diagnoses were verified from hospital case files based on the American College of Rheumatology (ACR)/European League Against Rheumatism (EULAR) 2010 criteria, or for some cases diagnosed before 2010, the ACR criteria from 1987^18, 19^. The exclusion criteria were uncertain RA diagnosis, and diagnosis of ankylosing spondylitis, psoriatic arthritis, juvenile idiopathic arthritis, or other inflammatory arthritis.

The HUNT study was approved by the Regional Committee for Medical and Health Research Ethics (REK), the Norwegian Data Inspectorate, and the National Directorate of Health. All participants gave written informed consent, and the study was performed in accordance with the Helsinki declaration. The HuLARS study was approved by REK (REK Midt 2009/661), and the Norwegian Data Inspectorate.

No previous data were available for power calculations. Estimating around 60,000 participants with complete data and an MI rate of 4% during follow-up, we would have 80% power to detect an association with hazard ratio (HR) = 1.12 for a GRS for CAD or RA, given alpha = 0.05 and no correlation with traditional CAD risk factors, which is supported by previous literature^20, 21^. This was deemed sufficient to perform the study.

Genotyping, imputation, and quality control of SNPs were performed by the Genomics core facility at NTNU—Norwegian University of Science and Technology on samples from 71,860 participants as described previously^22, 23^. For each sample, one of three HumanCoreExome arrays from Illumina Inc. (San Diego, CA, USA) were used, i.e. HumanCoreExome12 v1.0, HumanCoreExome12 v1.1, or UM HUNT Biobank v1.0. Exclusion criteria were as follows: samples not reaching a 99% call rate, or with contamination > 2.5% (as estimated with BAF Regress), large chromosomal copy number variants, lower call rate of a technical duplicate pair and twins, gonosomal constellations other than XX and XY, or where the inferred sex contradicted the reported gender. Genetic variants were evaluated for their genomic position and strand orientation using alignment of their probe sequences against the human genome from Genome Reference Consortium Human genome build 37 and the revised Cambridge Reference Sequence of the human mitochondrial DNA (https://genome.ucsc.edu). Variants with a call rate < 99% and participants of other than recent European ancestry were excluded. Imputation was performed using Minimac3 (v2.0.1)^23^ on a samples of recent European ancestry and a merged reference panel that was constructed by combining the Haplotype Reference Consortium panel (release version 1.1) and a local reference panel based on 2202 whole genome sequenced HUNT study participants. For the present study, SNPs for the main dataset, which had minor allele frequency of > 1% and did not deviate from Hardy–Weinberg equilibrium with significance below *p* = 10^–4^, were provided by the HUNT biobank. Each genetic risk variant for CAD and RA was weighted by the sum of risk alleles multiplied by the natural logarithm of their odds ratio from previous association studies to provide wGRS for RA and CAD for each participant.

Due to potential differences in populations and coverage of heritability, we evaluated six published CAD wGRS from three studies in the general population and finally included scores with 6 or 10 SNPs (from^24^), 157 SNPs (from^25^), and 25, 18, or 21 SNPs (from^26^). Some SNPs from the original scores were not available summarized in (Supplementary List S1). The RA wGRS were previously constructed based on an initial list of 269 SNPs reported from 30 association studies on RA in Caucasian populations as previously reported^13^. This former study showed that a smaller SNP selection was a better predictor for RA in HUNT than the complete selection^13^, but because the relationship of these wGRS to MI was not previously investigated, all six RA wGRS were included in the present study. None of the SNPs in the RA wGRS were also included in the CAD wGRS. As previously described^13^, the first wGRS was constructed using all these SNPs. The number of SNPs was then reduced using various selection schemes, resulting in five other wGRS. Firstly, 88 SNPs showing *p* values ≤ 5 × 10^–8^ and low LD on each chromosome in previous association studies were identified and used for construction of a second wGRS. The number was further reduced based on Lasso regression, resulting in 27 SNPs that were used for construction of a third wGRS. Using an alternative selection scheme starting with SNPs showing *p* values ≤ 5 × 10^–6^ in previous studies, 115 SNPs with low LD on each chromosome were identified and used for a fourth wGRS. Finally, Lasso regression was performed without prior selection, resulting in identification of 62 and 50 SNPs for the fifth and sixth wGRS^13^. All evaluated wGRS were standardized (swGRS) by their standard deviations in the dataset (N = 67,497; Fig. 1) to permit direct model comparison.

Baseline characteristics were compared between MI cases and controls using Student’s *t* test or the Mann–Whitney *U* test, and the Chi-square test. Data were analyzed using Cox proportional hazards regression. The endpoint was the first MI during follow-up, defined as a binary (yes/no) variable. The time variable was age in years, beginning when the person first participated in HUNT (either HUNT2 or HUNT3). Variables were updated when possible to account for potential differences in the participants’ traditional CAD risk factors, or RA disease status between HUNT2 and HUNT3. For those who participated in both HUNT2 and HUNT3 and had missing data for such time-varying covariates in HUNT3, we used their HUNT2 data. We excluded participants given an RA diagnosis after HUNT3 (n = 32). Censoring occurred on the date of first observed MI, the date of death, or 31.12.2017, whichever happened first.

**Figure 2:**
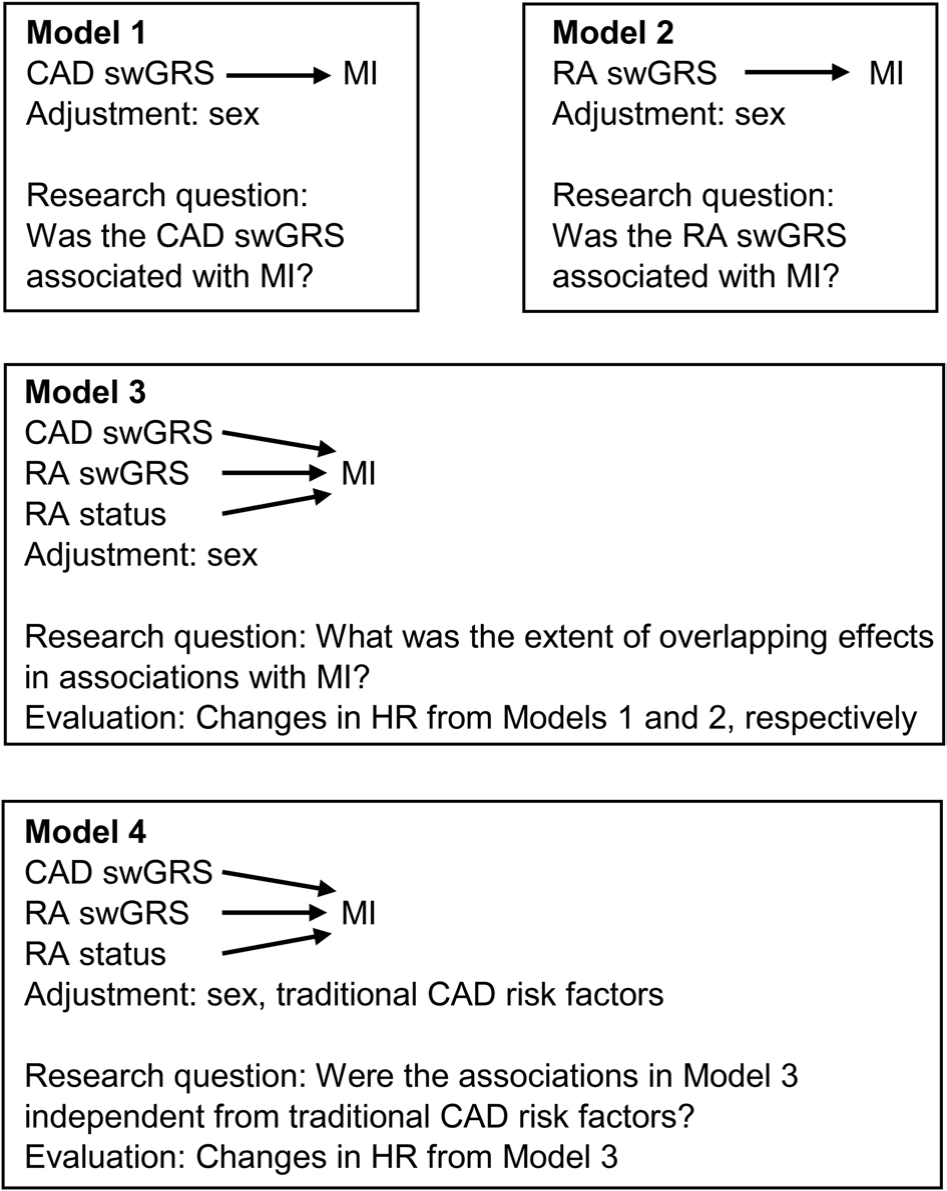
Cox regression analysis outline. Variables and research questions for the stepwise Cox regression analysis. Age was the time variable and thereby participants were compared with other participants of their own age. Traditional CAD risk factors in Model 4 were smoking, hypertension, total cholesterol, body mass index, self-reported previous cardiovascular disease, and diabetes. Abbreviations: CAD: coronary artery disease; MI: myocardial infarction; RA: rheumatoid arthritis; swGRS: standardized, weighted genetic risk score.

Each of the 6 CAD swGRS and 6 RA swGRS were initially evaluated in univariable Cox regression models in order to identify the one CAD swGRS and one RA swGRS with the highest HR for further assessment in the study. HR was chosen as selection criterion because it best indicates the relationship with the endpoint, i.e. MI. This analysis was not adjusted for multiple testing because such adjustment would not influence the HR. Pairwise high linkage disequilibrium (LD, i.e. r^2^ > 0.8) among the SNPs included in the best-fitting CAD swGRS and RA swGRS on each separate chromosome was evaluated using LDlink^27^. Any such SNPs would be kept in their respective GRS in line with the aim of investigating overlapping genetic associations with MI. Further analysis was performed in a stepwise fashion, as outlined in Fig. 2. First, the selected CAD swGRS and RA swGRS were tested in bivariable models adjusted for sex (Model 1 and 2) to account for sex differences in the development of CAD. Thereafter, a multivariable model combining the CAD swGRS, RA swGRS, RA status, and sex (Model 3), was run to evaluate the extent of overlapping effects in associations with MI between CAD swGRS, RA swGRS, and RA status. Finally, several traditional CAD risk factors were added (Model 4) to evaluate if the associations of the two swGRS and RA status with MI were influenced by traditional CAD risk factors. The final adjustment variables in addition to sex and RA status included smoking status (never, previous, or current smoker), hypertension (defined as “yes” if systolic blood pressure ≥ 140 mmHg and/or diastolic blood pressure ≥ 90 mmHg, or self-reported usage of medication for hypertension), total cholesterol, body mass index (BMI) (kg/m^2^), self-reported previous cardiovascular disease (MI, angina pectoris, or stroke), and diabetes.

To evaluate whether the effects of swGRS for CAD and RA were different in participants with and without RA, multiplicative interaction terms between each of swGRS and RA status were assessed. Another multiplicative interaction term evaluated the potential overlap between the CAD swGRS and RA swGRS. Biological interaction refers to the situation where the effect of two causative factors is larger than the sum of the separate effect of the factors. To evaluate such additive biological interaction among RA status, the CAD swGRS and the RA swGRS, the Rothman approach was used^28^. For these tests, the two risk scores were dichotomized, pooling the three lowest quartiles as reference and comparing with the highest risk quartile. The univariable effect of RA status on MI was also evaluated using the Kaplan–Meier method and the log-rank test. The sample size was too limited to evaluate the performance of the model among RA patients only (433 participants with RA, of whom 45 developed MI) or to analyse potential time trends in MI development. We also considered that there were too few males with RA [n = 142 (33%)] to perform sex-specific analyses of MI.

Model fit was evaluated using Cox-Snell residuals and log-minus-log plots. The proportional hazard assumption was evaluated using Schoenfeld residuals and log–log plot of survival. The functional form of continuous variables was evaluated by Martingale residuals. Models were compared using log likelihood, the Akaike (AIC) and Bayesian (BIC) information criteria. Stata (v.15, StataCorp, College Station, Texas, USA) was used for statistical analysis. *P* values < 0.05 were regarded statistically significant. Data are presented as mean ± SD or HR (95% CI) unless otherwise stated.

## Results

The initial dataset included 77,040 participants of Caucasian ancestry (578 RA cases; 76,462 controls) as in previous work^13^ (Fig. 1). After exclusion of participants due to incomplete genetic data and missing information on adjustment variables which we assumed was random, the final dataset included 61,465 participants of whom 2609 developed MI during the follow-up time (Fig. 1) and 433 had RA. Total time at risk was 5712 patient years for RA cases and 1,059,869 patient years for the controls. Among MI cases (n = 2609), the median time to event from baseline participation in HUNT was 11.3 years for RA patients (n = 58), and 15 years for controls (n = 2551) (*p* = 0.002). After exclusion of MI cases with self-reported previous cardiovascular disease, the median time to event was 11.4 years for RA patients (n = 48), and 15.2 years for controls (n = 2173) (*p* = 0.004).

Table 1 indicates that participants who experienced MI were comparatively older, were more often men and had a higher burden of traditional CAD risk factors. Furthermore, there was a significantly higher proportion of RA cases in the MI group.

**Table 1:** Baseline characteristics of study participants.

	Participants with baseline in HUNT2 (49,907)			Participants with baseline in HUNT3 (11,558)		
	No myocardial infarction (n = 48,151)	Myocardial infarction (n = 1756)	*P* value	No myocardial infarction (n = 10,705)	Myocardial infarction (n = 853)	*P* value
Rheumatoid arthritis, n (%)	345 (0.7)	37 (2.1)	< 0.001	43 (0.4)	8 (0.9)	0.02
Seropositive RA^a,b^	240 (73.2)	25 (73.5)	0.96	32 (74.4)	8 (100.0)	0.11
Age (years), mean ± SD	48 ± 16	56 ± 13	< 0.001	43 ± 16	68 ± 12	< 0.001
Men, n (%)	22,513 (46.8)	1110 (63.2)	< 0.001	4849 (45.3)	591 (69.3)	< 0.001
Previous cardiovascular disease^c^, n (%)	3131 (6.5)	249 (14.2)	< 0.001	463 (4.3)	655 (76.8)	< 0.001
Smoking			< 0.001			< 0.001
Previous smoker, n (%)	11,691 (24.3)	491 (28.0)		2867 (26.8)	416 (48.8)	
Current smoker, n (%)	14,714 (30.6)	661 (37.6)		2288 (21.4)	199 (23.3)	
Body mass index (kg/m^2^), mean ± SD	26.2 ± 4.0	27.2 ± 3.8	< 0.001	26.8 ± 4.7	28.2 ± 4.3	< 0.001
Hypertension^d^, n (%)	19,483 (40.5)	1061 (60.4)	< 0.001	2794 (26.1)	618 (72.5)	< 0.001
Diabetes mellitus, n (%)	1305 (2.7)	84 (4.8)	< 0.001	331 (3.1)	113 (13.3)	< 0.001
Total cholesterol, mean ± SD	5.8 ± 1.2	6.4 ± 1.2	< 0.001	5.2 ± 1.1	4.9 ± 1.3	< 0.001
HDL^e^ cholesterol, mean ± SD^b^	1.4 ± 0.4	1.3 ± 0.4	< 0.001	1.3 ± 0.3	1.2 ± 0.3	< 0.001
Triglycerides, mean ± SD^b^	1.7 ± 1.1	2.2 ± 1.3	< 0.001	1.6 ± 1.0	2.0 ± 1.2	< 0.001

Number of participants: 61,465. Number of MI cases: 2609, as indicated for final dataset (Fig. 1).

^a^Data missing for 20 observations at HUNT2 for RA seropositive; 18 observations at HUNT2, and 1 observation at HUNT3 for HDL cholesterol; 1 observation at HUNT2, and 1781 observation at HUNT3 for triglycerides.

^b^Seropositive RA: Positive for rheumatoid factor and/or anti-citrullinated protein antibody.

^c^Previous cardiovascular disease: Self-reported myocardial infarction, angina pectoris or stroke.

^d^Hypertension (defined as a “yes” if systolic blood pressure ≥ 140 mmHg and/or diastolic blood pressure ≥ 90 mmHg, or self-reported usage of medication for high blood pressure).

^e^HDL: high density lipoprotein.

Each of the six CAD swGRS were statistically significant in a univariable Cox model (Supplementary Table S1). Given its highest HR [1.23 (95% CI 1.19–1.28)] for each standard deviation increase), we chose the CAD swGRS based on 157 SNPs, hereafter called “CAD swGRS157” from^25^. Two of our previously constructed RA swGRS^13^ showed significance in a univariable Cox model. We selected the RA swGRS which was based on 27 SNPs, hereafter called “RA swGRS27” [HR = 1.04 (95% CI 1.00–1.08)] (Supplementary Table S1), as previously described^13^. We have previously shown that the heritability captured by RA swGRS27 was very similar to that captured by the largest RA swGRS, i.e. 5.2% vs. 5.3%^13^. Of the 157 SNPs included in CAD swGRS157, 23 (14.6%) were genotyped, and of the 27 SNPs included in RA swGRS27, 11 (40.7%) were genotyped. The remaining SNPs were imputed. The only SNPs included in either the RA swGRS27 or the CAD swGRS157 showing a pairwise high LD were rs4766578 and rs10774624 on chromosome 12, (r^2^ = 0.85).

CAD swGRS157 remained statistically significant in multivariable Cox regression models [HR = 1.23 (95% CI 1.18–1.27)] in model 4 (Table 2). RA swGRS27 was statistically significant [HR = 1.05 (95% CI 1.01–1.09), *p* = 0.02] in a multivariable Cox model including sex. However, RA swGRS27 became nominally non-significant with little numerical change in HR after inclusion of additional variables [HR in model 3 = 1.04 (95% CI 1.00–1.08), *p* = 0.07; HR in model 4 = 1.04 (95% CI 1.00–1.08), *p* = 0.06] (Table 2).

**Table 2:** Cox proportional hazards regression analysis for myocardial infarction.

	Hazard ratio (HR)	*P* value	95% CI for HR
**Model 1**			
Sex (male vs female)	2.28	< 0.0001	2.10,2.47
CAD swGRS157^a^	1.24	< 0.0001	1.20,1.29
**Model 2**			
Sex (male vs female)	2.26	< 0.0001	2.09,2.45
RA swGRS27^b^	1.05	0.02	1.01,1.09
**Model 3**			
RA status	2.17	< 0.0001	1.62,2.92
Sex (male vs female)	2.30	< 0.0001	2.12,2.49
CAD swGRS157^a^	1.24	< 0.0001	1.20,1.29
RA swGRS27^b^	1.04	0.07	1.00,1.08
**Model 4**			
RA status	2.08	< 0.0001	1.55,2.80
Sex (male vs female)	2.23	< 0.0001	2.05,2.42
CAD swGRS157^a^	1.23	< 0.0001	1.18,1.27
RA swGRS27^b^	1.04	0.06	1.00,1.08
Smoking status			
Previous smoker	1.09	0.10	0.98,1.20
Current smoker	1.83	< 0.0001	1.66,2.02
Hypertension	1.43	< 0.0001	1.30,1.56
Cholesterol (total)	1.09	< 0.0001	1.05,1.12
Body mass index	1.03	< 0.0001	1.02,1.04
Previous cardiovascular disease	1.63	< 0.0001	1.47,1.80
Diabetes	1.59	< 0.0001	1.38,1.82

Number of participants: 61,465. Number of MI cases: 2609, as indicated for final dataset (Fig. 1).

^a^CAD swGRS157: weighted genetic risk score for coronary artery disease based on 157 SNP used (Ref^25^), standardized using standard deviation in the present population.

^b^RA swGRS27: The best-fitting score for RA, developed previously (Ref^13^), standardized using standard deviation in the present population.

**Figure 3:**
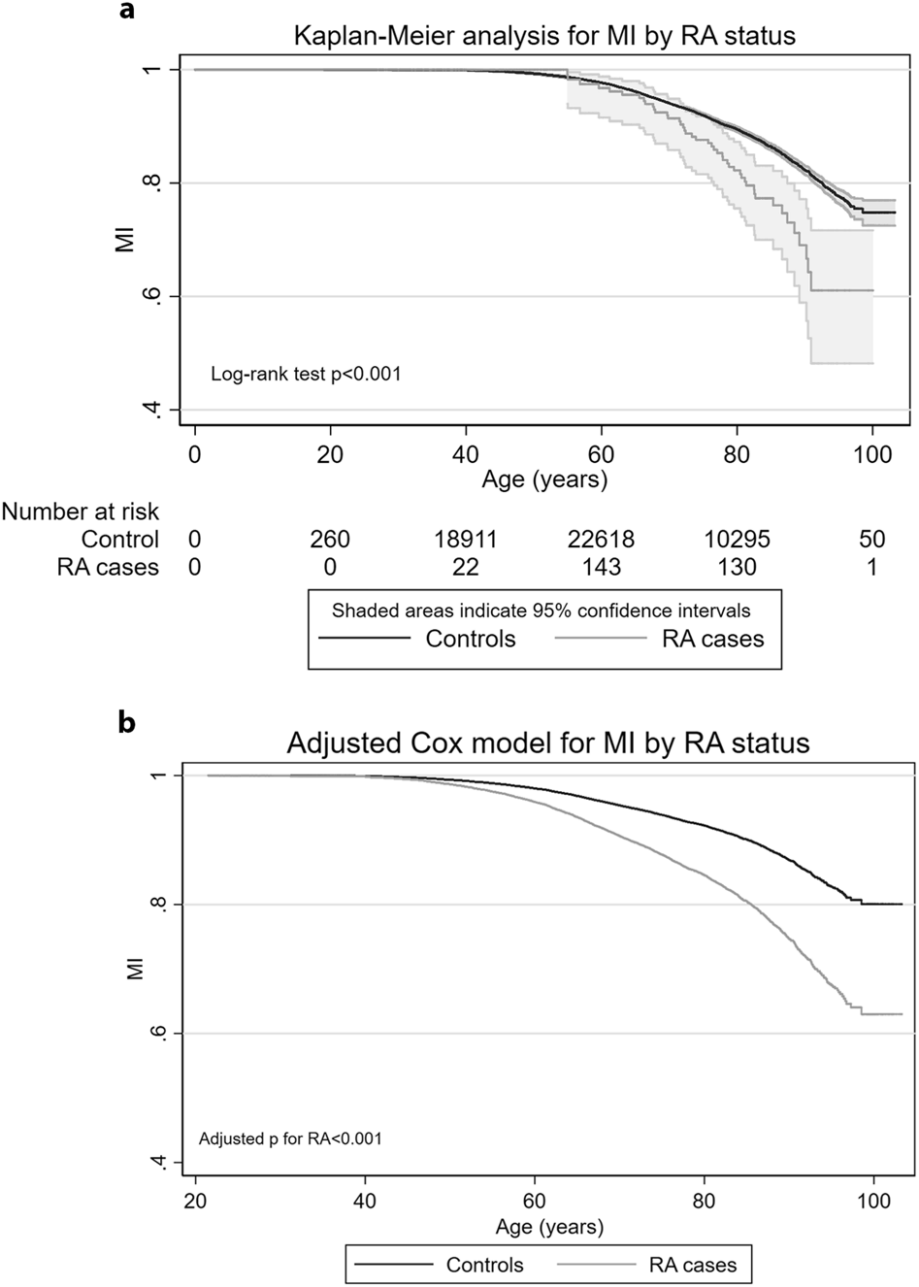
Estimates for myocardial infarction by RA status. Panel A: Kaplan–Meier curves, i.e. unadjusted model. Shaded areas indicate 95% confidence intervals. Panel B: Curves from Cox regression (Model 4, Table 2, adjusted for sex, CAD swGRS157, RA swGRS27, smoking, hypertension, total cholesterol, body mass index, previous cardiovascular disease, and diabetes). The analysis included 61,465 participants (2609 with and 58,856 without a myocardial infarction). Abbreviations: CAD: coronary artery disease; MI: myocardial infarction; RA: rheumatoid arthritis; swGRS: standardized, weighted genetic risk score.

RA status [HR = 2.08 (95% CI 1.55–2.80)] was significantly associated with an increased hazard of MI independently of traditional CAD risk variables, CAD swGRS157, and RA swGRS27 (Model 4, Table 2). The effect of RA status using the Kaplan–Meier method and Cox regression is illustrated in Fig. 3.

The interaction terms between RA status and either CAD swGRS157 (*p* = 0.47) or RA swGRS27 (*p* = 0.24) were non-significant when added to model 4 (Table 2). Furthermore, the interaction term between CAD swGRS157 and RA swGRS27 was non-significant (*p* = 0.40). As seen by the 95% CI overlapping zero, there were no significant biological interactions between RA status and either CAD swGRS157 [relative excess risk due to interaction − 0.29 (95% CI − 1.89, 1.31); attributable portion due to interaction − 0.12 (95% CI − 0.86, 0.62)] or RA swGRS27 [relative excess risk due to interaction − 0.21 (95% CI − 1.08, 1.50); attributable portion due to interaction 0.09 (95% CI − 0.43, 0.61)], or between CAD swGRS157 and RA swGRS27 (relative excess risk due to interaction 0.18 (95% CI − 0.08, 0.43); attributable portion due to interaction 0.11 (95% CI − 0.04, 0.26)].

Cox-Snell residuals showed acceptable overall fit for model 4 and there were no violations to the proportional hazards assumption. For models 1–4 in Table 2, the AIC were 49,167.98, 49,286.63, 49,146.76, and 48,716.64, and the BIC were 49,186.87, 49,305.52, 49,184.55, and 48,820.55, respectively. This implies best fit (lower AIC and BIC) for model 4 after inclusion of RA status, CAD swGRS157, and RA swGRS27, and the traditional CAD risk variables.

## Discussion

To our knowledge, this is the first study to evaluate the association of a wGRS for RA and a wGRS for CAD with MI in a large population-based cohort including RA patients and controls. Using Cox regression, the model was predictive for MI in the Norwegian HUNT cohort. The published wGRS for CAD^25^ was significantly associated with MI without significant statistical or biological interaction with RA status. However, the wGRS for RA did not reach nominal statistical significance, and there was little overlap in association between the CAD wsGRS and the RA wsGRS. The present study could be regarded as an external validation of several previously developed wGRS for CAD and provides evidence in favor of generalizability. Nevertheless, the predictive ability of present wGRS models is limited at an individual level.

A previous population-based study reported that a GRS based on 50 SNPs with genome-wide association with incident coronary heart disease (CHD), was significantly associated with CHD after adjustment for established risk factors^29^. Although some CAD genetic variants have shown association with traditional CAD risk factors^30^, most established CAD genetic risk variants are found in regulatory regions of the genome with unknown roles in CAD pathogenesis. They may be related to gene-environment interactions, rather than having independent effects in CAD development^31^. Most CAD genetic risk variants are considered independent of traditional CAD risk factors such as plasma lipids, diabetes, and hypertension but might be associated with atherosclerosis^20, 21^. Nevertheless, separating environmental and genetic factors in the development of complex diseases like CAD is problematic because current GWAS settings are more likely to ignore genetic risk variants that exert regulatory influences at particular prior environmental contexts, during shorter time intervals, and at later stages of CAD development^31^.

RA swGRS27 became non-significant in model 3 (*p* = 0.07), upon the inclusion of RA status and CAD swGRS157. This may be a false-negative conclusion due to the relatively low number of RA cases in the study population. A SNP included in RA swGRS27 (rs10774624) was in high LD with a SNP included in CAD swGRS157 (rs4766578). However, the overlapping effect on the risk of MI development was probably not strong enough to reach statistical significance in model 4. Furthermore, there were no significant interactions between RA swGRS27 and the variables RA status and CAD swGRS157 when tested in model 4. This might be due to low statistical power to properly identify interaction effects^32^. A study based on 61 genome-wide associated RA SNPs did not support their association as a group with CAD^33^. However, genetic variants associated with the development of CAD in RA patients and variants associated with both RA and CAD have been reported for selected loci in a genome-wide association study as well as candidate gene studies^14, 33–35^. Another factor that may have influenced our results is that genetic variants of relevance to inflammatory or immune responses which are considered determinative at later stages of atherosclerosis and CAD are less likely to be identified by current GWAS^31^.

Even after adjustment for other variables, RA status remained significant, implying that there are potential RA-specific characteristics with relevance to occurrence of MI that are not entirely explained by the included RA-related and CAD-related genetic variants and other traditional CAD risk factors. A study suggested that traditional cardiovascular risk factors and variables related to RA disease activity explained about 49% and 30%, respectively, (combined 70%) of the population-attributable CVD risk among RA patients^6^. Even if we had information on basic RA characteristics for our cases, such data were not available in the controls because of the population-based setting of HUNT and could therefore not be included in the analysis.

In line with our study, a prospective study also reported RA status as a statistically significant variable for CVD after adjustment for other mainly clinical variables^36^. Furthermore, a meta-analysis concluded that traditional cardiovascular risk factors are independently associated with CVD among RA patients as in the general population^37^.

The study has some limitations. The endpoint definitions may have been slightly different between previously published risk scores^24–26^ and the current study. For some participants, the endpoint may not have been their first MI. Identification of MI might be different among RA cases and controls due to surveillance bias and risk of silent MI in RA patients^38^. Persons with more active RA may also be less likely to participate in HUNT. Another challenge is related to the potential effect of RA therapy on the incidence of CAD^12^ which could be accounted for in future studies. We used self-reported data for previous CAD, which could introduce bias.

The study also has several strengths. One of the major strengths was the prospective population-based setting with a relatively large unselected cohort including RA patients and controls from the same population. In addition, minimal right censoring occurred due to loss of follow-up and death during follow-up was accounted for. The hospital registry includes almost every MI of the county. However, a few participants may have emigrated or had their MI abroad without local follow-up in Trøndelag. The influence on our results was probably small.

In conclusion, a combined model based on traditional CAD risk factors, RA status, and swGRS for CAD and RA was predictive for MI in a Norwegian cohort. Although the swGRS for CAD was relevant for MI, the SNPs included in the present swGRS for RA did not seem to contribute strongly to the risk for MI.

## Supplementary information

Supplementary Information.

## Data Availability

Data from the HUNT Study are available upon reasonable request from the HUNT Research Centre (https://www.ntnu.edu/hunt/data), following approval from the Regional Research Ethics Committee. However, restrictions apply to the availability of the data for the present paper, which were used under license for the current study and are not publicly available, in accordance with Norwegian law.
