# Supplementary material for: Genetic risk score associations for myocardial infarction are comparable in persons with and without rheumatoid arthritis: the population-based HUNT study
Source: Sci Rep. 2020 Nov 24;10:20416. doi: 10.1038/s41598-020-77432-0 (PMC7686351; doi:10.1038/s41598-020-77432-0)
Supplement: Supplementary file 1 — Supplementary Information. [file 41598_2020_77432_MOESM1_ESM.pdf]

Additional file 1 Supplementary information

Manuscript title: Genetic risk score associations for myocardial infarction are comparable in persons with and without rheumatoid arthritis – the population-based HUNT study

S. Rostami, M. Hoff, H. Dalen, K. Hveem, V. Videm

Supplementary List S1: Genetic risk variants excluded from each genetic risk score for coronary artery disease employed in the present study

For the wGRS based on 25 and 21 SNPs from (Ref 26), all SNPs were available. For the wGRS based on 8 and 12 SNPs from (Ref 24), 182 SNPs from (Ref 25), and 19 SNPs from (Ref 26), some SNPs were not available. The excluded SNPs included were insertions/deletions (marked as I or D below), SNPs that were not genotyped or imputed and where no proxy SNPs were available, and variants covered by Norwegian law regarding ethical guidelines for strong determinants of defined medical conditions.

Excluded SNPs:
From reference 24
GRS8: rs67258870; rs9982601
GRS12: rs67258870; rs9982601

From reference 25
GRS182: rs142695226; rs6876322; rs115696548; rs7212798; rs180803; rs56062135; rs56289821; rs6511721; rs28451064; rs7280276; chr6:160776695:I; chr4:156366138:I; chr19:41790086:D; chr3:138099161:I; chr12:125209562:I; chr8:142230002:D; chr3:172117455:D; chr14:75614504:I; chr4:82625720:D; chr16:75308440:D; chr6:160265331:D; chr10:75595440:D; chr19:45801579:D; chr1:110299165:I; chr6:12619932:D

From reference 26
GRS19: rs17228212

Supplementary Table S1. Univariable Cox regression for the standardized weighted genetic risk scores for coronary artery disease (CAD) and rheumatoid arthritis (RA)[a], with myocardial infarction as endpoint

	Hazard Ratio	P-value	95% CI for Hazard Ratio
**CAD risk scores[b]**			
CAD swGRS6	1.18	<0.0001	1.13,1.22
CAD swGRS10	1.17	<0.0001	1.12,1.21
CAD swGRS157	1.23	<0.0001	1.19,1.28
CAD swGRS25	1.16	<0.0001	1.11,1.20
CAD swGRS18	1.20	<0.0001	1.16,1.25
CAD swGRS21	1.20	<0.0001	1.16,1.25
**RA risk scores[c]**			
RA swGRS269	1.01	0.76	0.97,1.05
RA swGRS62	1.03	0.08	1.00,1.08
RA swGRS50	1.04	0.05	1.00,1.08
RA swGRS115	1.01	0.77	0.97,1.05
RA swGRS88	1.02	0.24	0.98,1.06
RA swGRS27	1.04	0.04	1.00,1.08

[a]Weighted risk scores standardized for the corresponding standard deviation in the dataset of 2,828 MI cases and 64,669 controls (Figure 1).
[b]CAD risk scores from published studies (List S1).
[c]RA risk scores previously constructed (Ref 13).
